# CrossFit^®^ and Its Influence on Health Behaviors, Functional Capacity, and Psychosocial Outcomes: An Explorative Study of Gender Differences in Athlete Perspectives

**DOI:** 10.3390/jfmk10020196

**Published:** 2025-05-28

**Authors:** Alessandra Amato, Luca Petrigna, Leonardo Di Gregorio, Giuseppe Musumeci

**Affiliations:** 1 Department of Biomedical and Biotechnological Sciences, Section of Anatomy, Histology and Movement Science, School of Medicine, University of Catania, Via S. Sofia n°97, 95123 Catania, Italy; alessandra.amato@unict.it (A.A.); luca.petrigna@unict.it (L.P.); leonardodigregorio01@gmail.com (L.D.G.); 2 Research Center on Motor Activities (CRAM), University of Catania, Via S. Sofia n°97, 95123 Catania, Italy

**Keywords:** sport, functional training, wellness, performance, body composition, lifestyle

## Abstract

**Objectives:** This explorative study aimed to evaluate athletes’ perceptions of how CrossFit^®^ affects physical, psychological, and social well-being, exploring gender differences. CrossFit^®^ is a high-intensity functional training modality aimed at enhancing overall fitness and health. Limited research has explored its perceived impact on broader aspects of well-being, particularly considering gender differences. **Methods**: A total of 202 participants (age 34.3 ± 10.0 years) with at least 6 months of CrossFit^®^ experience completed an online self-reported questionnaire not previously published but created following published guidelines. The questions explore athletes’ perceptions of their improvements in physical fitness, injury occurrence, and the effects on mental well-being. Statistical analysis included descriptive statistics and parametric and non-parametric tests to investigate gender differences. **Results**: In total, 81.2% of participants perceived improvements in strength, while 83.2% reported enhanced aerobic capacity. Significant body composition improvements were noted by 68.3% of the sample, and 87.6% reported changes in eating habits. Injury incidence was lower among women (30.2%) than men (45.3%) (*p* = 0.02). Regarding psychological outcomes, 95% of respondents indicated a reduction in stress levels, 73.3% reported better sleep quality, and over 90% perceived improvements in self-esteem and daily energy. Furthermore, 98.5% of participants formed new social connections through CrossFit^®^, with 79.2% acknowledging a positive impact on social relationships. No significant gender differences were found across most psychological and social outcomes. **Conclusions**: CrossFit^®^ is perceived positively by participants, with similar views across genders, except for injury rates. It enhances physical fitness, mental health, and social well-being, with high training adherence and relatively low injury risk.

## 1. Introduction

CrossFit^®^ (CrossFit, Inc., Washington, DC, USA) is a form of functional fitness training (FFT) that falls under the broader category of high-intensity interval training (HIIT), and it is recognized as one of the main modalities of high-intensity functional training (HIFT) [1]. It is based on functional movements, which are universal motor patterns that replicate daily life activities, combined into a program designed to develop physical capacities across ten fitness domains: cardiovascular and respiratory endurance, strength, flexibility, power, speed, coordination, agility, balance, and accuracy [2]. CrossFit^®^ merges military-style training with aerobic and gymnastic exercises, attracting individuals seeking fast results in fat loss and an alternative to traditional gym routines. Workouts are typically structured as high-intensity circuits with little to no rest between exercises, which often include high-repetition lifts of barbells and dumbbells. This intense format, especially when performed without proper supervision or technique, increases the risk of musculoskeletal injuries [3,4]. Over the past decade, CrossFit^®^ has experienced exponential growth, with millions of practitioners and affiliated training centers worldwide. It has been consistently ranked among the top global fitness trends by the American College of Sports Medicine (ACSM) [5]. While the scientific literature on CrossFit^®^ has expanded, focusing on its effects on physical performance (e.g., strength, endurance, and body composition) [6], physiological adaptations, and injury risk, limited research has explored athletes’ perceptions of its impact on broader aspects of well-being, such as stress, sleep, and social relationships.

Notably, CrossFit^®^ is widely recognized for fostering a strong sense of community and motivational support, which may contribute positively to psychosocial well-being. The link between social relationships and overall health is well established [7]. According to the World Health Organization, well-being is defined as “a state of complete physical, mental, and social well-being, and not merely the absence of disease or infirmity” [8]. Social support has been associated with biological markers of health, including reduced inflammation and slower cellular aging, while low social support has been linked to increased inflammatory activity and stress responses [9]. Therefore, exploring how CrossFit^®^ influences social connections may provide valuable insights into its role in supporting overall well-being.

Sleep quality, another critical component of health, is also relevant in this context. Poor sleep is associated with a higher risk of illness, injury, and chronic disease, and it plays a crucial role in athletic performance and recovery [10]. Athletes’ sleep patterns are influenced by both sport-specific (e.g., training load, travel, and competition) and non-sport factors (e.g., psychological stress and gender). Stress itself is a key element in mental health, defined as a state of perceived or real threat to homeostasis. Chronic or poorly managed stress responses can contribute to the development of various physical and mental health conditions, particularly when exposure occurs during sensitive developmental periods such as prenatal life, childhood, and adolescence [11].

Despite growing interest in CrossFit^®^, gender differences in how individuals respond to and perceive this discipline remain underexplored. Existing studies tend to focus on performance outcomes, often highlighting strength and power differences between men and women. This performance gap may be exacerbated by the lack of adapted standards in gymnastics and metabolic conditioning exercises [12,13]. Mangine et al. (2023) documented that men typically complete repetitions at a faster rate than women in the CrossFit^®^ Open workouts, especially in gymnastics components, demonstrating greater pacing consistency and speed [14]. Another comparative analysis of the 2021 CrossFit^®^ Games showed that despite scaling standards for women, men outperformed women in 14 out of 15 events. This highlights potential limitations in how scaling is applied, particularly when gymnastics and monostructural conditioning exercises lack sex-specific adaptations. These discrepancies may contribute to divergent experiences and perceptions between male and female athletes [13].

However, very few studies have directly explored how CrossFit^®^ impacts lifestyle, physical functioning, and psychosocial well-being from a sex-specific perspective. One notable exception is the longitudinal study by Poderoso et al. (2019), which found significant gender differences in chronic hormonal and immune responses over six months of CrossFit^®^ training. Men experienced increases in testosterone and greater reductions in cortisol compared to women, suggesting sex-specific physiological adaptations that may also affect subjective experience and overall well-being, although this was not explicitly examined [15]. Furthermore, Sugimoto et al. (2020) reported that female CrossFit^®^ athletes were more prone to lower extremity injuries, while males were more likely to sustain shoulder injuries. Although the focus was clinical, these findings imply possible sex-based differences in risk perception, movement patterns, and self-efficacy, all of which could influence the psychological and lifestyle-related experience of the discipline [12].

These limitations, such as a lack of sex-stratified analyses, a narrow focus on performance, and a limited exploration of lifestyle and well-being, highlight a clear knowledge gap. A more detailed understanding of sex-specific experiences in CrossFit^®^ could support more inclusive programming and health promotion strategies.

This study aims to explore how athletes perceive the impact of CrossFit^®^ on multiple dimensions of well-being, with particular attention to potential gender differences. By understanding these perceptions, we hope to assess the sustainability of CrossFit^®^ as a long-term training approach and contribute to broader health promotion goals, aligning with recommendations from organizations such as the ACSM to support lifelong physical activity.

## 2. Materials and Methods

### 2.1. Study Design

All athletes were invited to fill out a self-reported online questionnaire presented to them via a link to “Google^®^ forms”. The participants were informed about the aim of the study, and they provided their written informed consent. The study was approved by the Research Center on Motor Activities (CRAM) Ethics Committee of the University of Catania (Protocol no.: CRAM-030-2023, 15 March 2023). The study followed the principles of the Declaration of Helsinki.

### 2.2. Participants

Participants were recruited using a convenience sampling strategy, which involves selecting individuals who are readily accessible and willing to participate. They were reached in person by coaches during training time in the CrossFit^®^ box or contacted by telephone number, social media, and by email within the local community (Sicily, Italy).

The inclusion criteria were the practice CrossFit^®^ for at least six months, being over 18 years old, being familiar with digital devices for filling out the questionnaire, and having signed the informed consent form. The participants had to be currently practicing CrossFit. The non-occurrence of one of these elements was considered an exclusion criterion.

### 2.3. Self-Reported Questionnaire

The self-reported online questionnaire was proposed in the Italian language. It was not previously published or validated. When the questionnaire was written, guidelines were followed [16]. After the determination of the topic, the questions were written and proposed to colleagues to understand if the questionnaire was easy to understand, clear, and feasible. A simple language was adopted, and the number of questions was chosen so the questionnaire could be completed in about ten minutes. The first section is characterized by a description of the goal of the questionnaire/study (the cover sheet). The details of the principal investigator were provided, and it was clearly written to ask if there were questions or doubts related to the study. In this section, the informed consent and the modality of the data treatment (confidential and anonymous) were also provided. It was also written that the participants could withdraw the questionnaire at any moment without penalty. To confirm their will to participate in the questionnaire, the participants had to tape their name and surname after this section. In the first part of the second section, information related to personal data, anthropometric measurements, and training habits were collected. In the second part, we aimed to analyze athletes’ perceptions of strength and endurance and body composition improvements, injury incidence, and impacts on psychological well-being, including work productivity and social relationships. The section included 15 questions; there were two types of response scales: multiple-choice options and free-response questions.

- To assess athletes’ perceptions of physical performance improvements, the sample was asked to answer the following questions:

(1) “Have you noticed an improvement in your physical strength since you started practicing CrossFit^®^?”, choosing from 4 answer options: “very much improved”, “moderately improved”, “slightly improved”, and “no improvement”; (2) “Have you seen any improvement in the 1RM back squat?”, with the answer options “yes” or “no”; and (3) “Have you seen any improvements in the 2 km row?”, with the answer options “yes” or “no”.

- To assess athletes’ perceptions of body composition improvements, the sample was asked to answer the following questions:

(4) “Have you lost weight or body fat since you started CrossFit^®^?”, with the answer options being “yes, considerably”, “yes, moderately”, “yes slightly”, and “no”; and (5) “Have you rate the weight loss that justifies the previous answer? If yes, which ones?”, for which the answer options were “scales”, “bioimpedance analysis”, “plicometry”, and “other”.

- To quantify the injury incidence in CrossFit^®^ for the included sample, the following question was proposed:

(6) “Have you suffered injuries because of practicing CrossFit^®^?”, having the answer options “yes” or “no”.

- To understand the impact of CrossFit^®^ on psychological well-being, we presented the following questions:

(7) “Did CrossFit^®^ reduce your stress level?”, with possible answers of “very small”, “moderately reduced”, “slightly reduced”, and “no impact”; (8) “How would you rate the impact of CrossFit^®^ on your stress level?”, with the possible answers “very reduced”, “moderately reduced”, “slightly reduced”, and “no impact”; (9) “Have you noticed an improvement in your sleep quality (REM and deep sleep) since you started doing CrossFit^®^?”, with the possible answers “much improved”, “moderately improved”, “slightly improved”, and “no change”; (10) “Have you noticed a change in your self-esteem since you started practicing CrossFit^®^?”; (11) “How would you describe your daily energy level since you practice CrossFit^®^?” (the possible answer to the last two questions was the same: “very improved”, “moderately improved”, “little improved”, and “no change”); (12) “Has CrossFit^®^ affected your productivity at work or in the study?”, with possible answers of “yes in a positive way”, “yes in a negative way”, and “no impact”; (13) “Has CrossFit^®^ affected your social relationships?”, with the related answers being “yes, in a positive way”, “yes in a negative way”, and “no impact”; and (14) “Have you made new friends or met new people through CrossFit^®^?”, with the options “yes” or “no”.

- To understand athletes’ general opinions on CrossFit^®^, the following question was asked to understand the general positive or negative participants’ opinions on CrossFit^®^ participation:

(15) “Would you recommend CrossFit^®^ to other people?”

### 2.4. Statistical Analysis

Data were evaluated through descriptive statistics (means, standard deviations, and percentages). The a priori sample size was determined based on Cohen’s statistical power using chi-square tests [17]. An expected medium effect size, Cohen’s w = 0.30, was calculated based on the results of a similar previously published study [12]. The pwr.chisq.test() function from the pwr package in R (RStudio 2021.09.2 Build 382), assuming a significance level (α) of 0.05, power of 0.90, and 1 degree of freedom (comparison between male and female groups), was used. The result indicated that a minimum total sample size of about 117 participants would be required to detect a medium effect. The Shapiro–Wilk test was used to assess the normality of distribution for continuous variables to determine the appropriate use of parametric or non-parametric statistical tests. According to a normality check, the U Mann–Whitney test was used to analyze potential gender group differences for the continuous variables at a significance level (α) of 0.05. A non-parametric chi-square test was used to analyze potential gender group differences in questionnaire answers that include nominal variables. In addition, a binary logistic regression was conducted to assess predictors of variables that were significant in the chi-square test. Independent variables included the gender, age, training frequency, and categorized CrossFit experience levels. Odds Ratios (ORs), 95% confidence intervals, and *p*-values were reported. The statistical analysis was performed through Jamovi software (The Jamovi project, 2022; Jamovi Version 2.3 [Computer Software]; retrieved from https://www.jamovi.org (accessed on 21 April 2025)).

## 3. Results

### 3.1. Participant Characteristics

**Figure 1:**
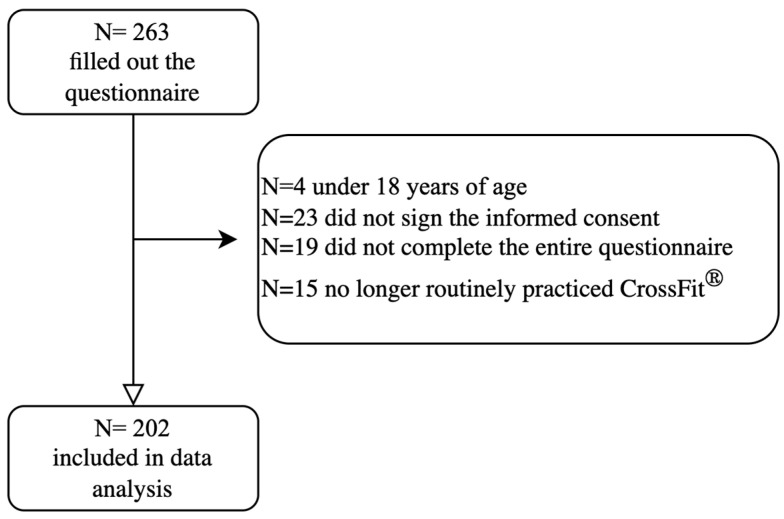
Flowchart of participant selection. Of the 263 individuals who completed the questionnaire, 61 were excluded due to the following reasons: under 18 years of age (N = 4), lack of signed informed consent form (N = 23), incomplete questionnaire (N = 19), or no longer routinely practicing CrossFit (N = 15). A total of 202 participants were included in the final data analysis (N = number of participants).

Of 263, 202 participants were included in the data analysis (Figure 1), with 47% females and 53% males, aged 34.30 ± 10.04 years, with a weight of 71.78 ± 13.21 kg and height of 169.96 ± 9.27 cm. In total, 49% of the sample carried out a sedentary job, followed by 39.6% who carried out a standing job, with the remaining 9.4% having a manual job and 2% having a heavy manual job; 37.6% of the sample dedicated an hour to housework activities. The majority of our sample (69.8%) have been practicing CrossFit^®^ for more than 24 months, 9.4% for 24 months, 7.9% for 12 months, and 12.9% for at least for 6 months. A total of 35.1% of the participants practice CrossFit^®^ four times a week, 25.2% practice five times a week, 19.3% practice three times a week, 15.3% train six times a week, 2.5% train two times a week, and 1% practice seven times a week. As for the hours spent training, 55.9% of the participants said they dedicated one hour to training, 31.7% of the participants spent two hours training, 9.4% spent three hours training, and only 3% of the participants trained for more than four hours. Regarding participation in official CrossFit^®^ competitions, 57.4% of the participants stated that they had never participated in official competitions, and 42.6% stated that they had. The sample analyzed had an average weekly CrossFit^®^ training frequency of 4.30 ± 1.14 times, with an average of 95.6 ± 42.4 min per session.

The only statistically significant differences between men and women in the sample characteristics were in their body composition (weight: *p* < 0.05; height: *p* < 0.05; BMI: *p* < 0.05) and housework activities (*p* < 0.05). Table 1 presents the anthropometric and sociodemographic information of the participants divided by gender. These data were extrapolated from the first part of the questionnaire, as described in the Section 2.

### 3.2. CrossFit^®^ Practice Effect

In total, 81.2% of the sample declared an improvement in strength skills, and 96% of cases were detected by increasing the 1RM back squat. Instead, 83.2% of the sample demonstrated an improvement in their aerobic capacity by increasing the test in a 2 km row. Also, 36.1% of the sample declared a significant improvement and 32.2% a moderate improvement in body composition by evaluating their weight and fat percentage with scales or bioimpedance analysis. In addition, 87.6% of the sample changed their eating habits, the latter including various areas of nutrition, with the main one being to rely on a nutritionist. A total of 61.9% of the recruited sample stated having never had an injury performing CrossFit^®^ training. Also, 43.1% of the sample had a very important reduction in stress levels. Moreover, 38.6% had a moderate improvement in sleep quality (taking into consideration the REM phase and the deep sleep phase, using smartwatches), 26.7% say they had not noticed any kind of improvement, and 20.3% say they had greatly improved sleep quality. The practice of CrossFit^®^ also had an important impact on self-esteem; as many as 50% of the sample said they had a significant improvement, and 33.2% said they had a moderate improvement in their self-esteem. Energy levels were significantly increased in 45% of the sample and moderately improved in 39.6%, also affecting productivity at work, where 61.9% of the sample said they were positively influenced, while 34.2% did not notice any kind of impact. A strong influence on social relationships was recorded, confirmed by almost the entire sample, where 98.5% said that the practice of CrossFit^®^ has allowed them to meet new people. As far as the influence of relationships in general is concerned, 79.2% of the sample stated a positive influence. Table 2 shows the results for each question for the entire sample.

### 3.3. Gender Comparison

**Figure 2:**
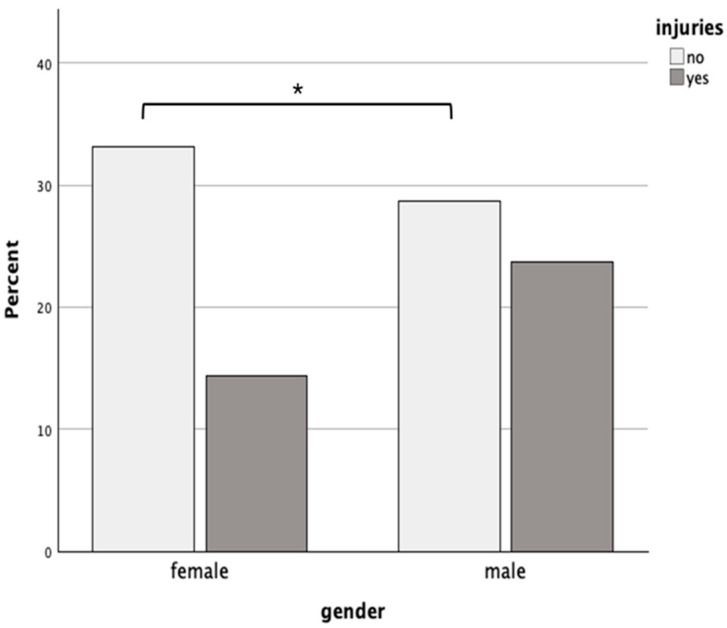
Bar graph representing the association via chi-square test between gender on the awareness “Have you suffered any injuries because of practicing CrossFit?”. The x-axis represents the gender, and the y-axis represents the frequency of responses. Dark gray depicts the ratio of participants who suffered injuries, and light gray depicts the ratio of participants who did not suffer injuries. The statistical difference between the groups was significant (* chi-square test, *p*-value = 0.02).

It was found that women were significantly less likely to experience injuries from practicing CrossFit^®^ than men X^2^ (N = 202, df = 1) 4.85, *p* = 0.03. Specifically, 30.2% of females reported experiencing an injury related to CrossFit^®^ practice compared to 45.3% of males who reported experiencing an injury (Figure 2).

In addition, logistic regression analysis revealed that the male gender (OR = 1.95; *p* = 0.039) and training experience over 24 months (OR = 4.36; *p* = 0.037) were significant predictors of injury. A trend toward decreased injury risk with an increasing age was observed (OR = 0.97; *p* = 0.063), although it was not statistically significant. Other variables, such as training frequency, were not significantly associated with injury risk.

There were no significant differences in the responses between the two genders for the other questions in the questionnaire. In particular, regarding the improvements found in physical function, 96.8% of women and 99.8% of men found an improvement in 1RM: X^2^ (N = 199, df = 1) 0.35, *p* > 0.05; 80.2% of women and 88.2% of men improved their performance in the 2 km row: X^2^ (N = 202, df = 1) 1.15, *p* > 0.05; and finally, 81.3% of women and 81.1% of men found their physical strength “much improved” since practicing CrossFit^®^: X^2^ (N = 202, df = 1) 0.00, *p* > 0.05. In terms of body perception, only 12.6 percent of women and 9.4 percent of men said they had not lost weight or body fat by practicing CrossFit^®^: X^2^ (N = 201, df = 3) 2.77, *p* > 0.05, and 16.7 percent of women and 8.5 percent of men said they had not changed their eating habits since practicing CrossFit^®^: X^2^ (N = 202, df = 1) 3.20, *p* > 0.05. Answers between males and females were also similar in their perceptions of CrossFit^®^’s impact on their psychological well-being:

In total, 95.8% of women and 89.6% of men said that practicing CrossFit^®^ reduced their stress levels: X^2^ (N = 202, df = 1) 2.83, *p* > 0.05; 72.9% of women and 73.6% of men noted improved sleep quality: X^2^ (N = 202, df = 1) 0.01, *p* > 0.05, with 93.8% of women and 96.2% of men also affirming an increase in perceived daily energy levels: X^2^ (N = 202, df = 1) 0.66, *p* > 0.05. In addition, 94.8% of women and 94.3% of men noted an increase in self-esteem: X^2^ (N = 202, df = 1) 0.02, *p* > 0.05. Regarding social relationships, 82.1% of women and 75.5% of men said that CrossFit^®^ positively influenced their social relationships: X^2^ (N = 202, df = 2) 2.18, *p* > 0.05. In particular, 97.9% of women and 99.1% of men made new friends because of practicing CrossFit^®^: X^2^ (N = 202, df = 1) 0.44, *p* > 0.05; finally, CrossFit^®^ positively influenced productivity at work in 63.5% of the female sample and 60.4% of the male sample: X^2^ (N = 202, df = 2) 0.44, *p* > 0.05.

**Figure 3:**
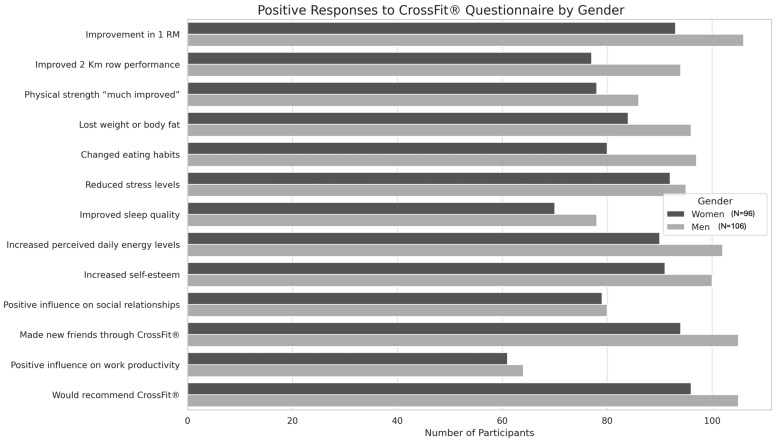
Positive responses to the CrossFit^®^ questionnaire by gender. The graph shows the number of male (N = 106) and female (N = 96) participants who reported improvements or positive effects in various areas because of their CrossFit^®^ practice. Areas assessed include physical performance (e.g., 1RM and rowing), body composition, eating habits, psychological well-being, social relationships, and work productivity. Overall, both genders reported high levels of perceived benefit, with slight variations in specific domains (*p* > 0.05).

In the final question about participants’ general opinion of CrossFit^®^, only one male participant answered that he would not recommend this discipline. Figure 3 summarizes the results described by emphasizing the number of male and female participants who gave positive responses for each question.

## 4. Discussion

The main objective of this study was to examine the impact of CrossFit^®^ on the lifestyle of an adult population, with a particular focus on possible gender differences in the perceived effects of this discipline across various dimensions of well-being. The results provide new insights into how CrossFit^®^ influences lifestyle, physical performance, body composition, psychological well-being, social relationships, and injury incidence. A key finding concerns training frequency. Participants reported engaging in CrossFit^®^ 4.30 ± 1.14 times per week, with an average session lasting 95.6 ± 42.4 min. These levels not only meet but exceed the American College of Sports Medicine (ACSM) guidelines [18], which recommend at least 150 min per week of moderate or 75 min of vigorous activity, or a combination thereof, to achieve optimal health benefits [19]. This suggests that for many individuals, CrossFit^®^ has become a stable and consistent component of their weekly routine. These findings are consistent with the literature indicating higher adherence and retention rates among CrossFit^®^ practitioners compared to those in traditional aerobic or resistance training programs [20].

### 4.1. Physical Function

Most participants reported improvements in strength and anaerobic capacity. These outcomes align with previous research, such as the meta-analysis by Wang et al., which found that high-intensity functional training (HIFT)—the basis of CrossFit^®^—enhances upper and lower body strength [21]. High-intensity functional training promotes musculoskeletal and metabolic adaptations by increasing both muscle fiber quality and cardiovascular efficiency, ultimately improving endurance and lactate tolerance. The repeated exposure to short, intense aerobic intervals typical of CrossFit^®^ contributes to improved cardiovascular adaptability and delayed lactate accumulation [22,23,24]. Regarding body composition, 68.3% of participants reported reductions in weight or fat mass. This is particularly relevant given the well-established link between excess body weight and a higher risk of chronic diseases. Exercise programs like CrossFit^®^, when paired with proper nutrition, can play a key role in obesity prevention [25]. However, the efficacy of such programs depends on the specificity of the training protocol, particularly the frequency, intensity, time, and type parameters. For instance, the study by Dehghanzadeh Suraki et al. showed significant reductions in weight, body mass index, and body fat after four weeks of five CrossFit^®^ sessions per week [26]. On the other hand, Aravena-Sagardia et al. showed that four weeks, with a total of 12 sessions conducted 3 times per week of the CrossFit^®^ training program, did not significantly change participants’ body weight, height, body mass index, and fat mass [27]. In Dehghanzadeh Suraki’s study, the participants, who started from an overweight condition in the Aravena-Sagardia et al. study, instead, were young and had normal weight. These findings suggest that the effectiveness of CrossFit^®^ on body composition may vary depending on the CrossFit^®^ protocol but also on the starting condition of the participants, who, in our study, had an average body mass index of 24.67 ± 2.77.

Injury rates were relatively low, with 61.9% of participants reporting no injuries related to CrossFit^®^ practice. These data are consistent with previous studies, such as a four-year investigation where 69.5% of participants reported no injuries. Notably, injuries were more common among those training fewer than three times per week, underscoring the importance of regular practice and adequate adaptation [28]. A recent systematic review by Gardiner et al. highlighted the shoulder, spine, and knee as the most injury-prone areas, with an overall injury rate ranging from 0.27 to 3.3 per 1000 training hours, which is comparable or even lower than in many other sports. However, the risk appears to increase with training frequency, competition involvement, and a lack of experience, reinforcing the need for attention to technique and progressive load increases [29]. Thus, comparing our results with those in the literature suggests that despite the demanding nature of CrossFit^®^, the overall injury risk appears moderate to low, and CrossFit^®^ is perceived as a safe activity for most participants. However, special attention should be paid to training volume, beginner athletes, and exercise execution during competition.

### 4.2. Psychosocial Well-Being

The positive impact on the sample’s social relationships shown in our results reflects the social and community role of CrossFit^®^, which, through the interaction between participants during training sessions, seems to promote new social networks. It has already been shown that a greater sense of community characterized CrossFit^®^ sessions when compared to traditional training [30]. However, CrossFit^®^’s motivational characteristics, which aim to lead the individual to achieve the best performance possible, could turn into negative attitudes, such as injuries and losses in social relations, because of overexercise, generating a prevalence of exercise addiction in CrossFit^®^ athletes associated with exercise despite injury, addiction turning into obsession, and taking medication to be able to exercise [31,32]. In addition, the participants positively rated the impact of CrossFit^®^ on stress levels, self-esteem, and sleep quality, aspects that could be associated with both the intensity of training and the reduction in perceived stress. These findings are in line with evidence linking regular physical activity with better stress management and higher sleep quality, particularly concerning deep sleep and the REM phase [33]. Along with diet and physical activity, sleep is an essential activity that plays a crucial role in emotional and physical development, health, and well-being. Quality sleep is associated with positive health outcomes such as attention, memory, and cognition improvement that directly affect the quality of life [33].

### 4.3. Analysis of Gender Differences

Our data indicate that men reported significantly more injuries than women (45.3% vs. 30.2%). This result aligns with previous research showing that male CrossFit^®^ athletes are more prone to shoulder injuries, whereas female athletes are more likely to sustain lower limb injuries [12]. These findings support the need for greater attention to execution technique and load progression to reduce injury risk, particularly for male athletes. The inclusion of logistic regression allowed for a more comprehensive evaluation of injury risk, confirming the male gender as an independent risk factor and identifying longer training experience as a potential contributor to increased injury risk. This multivariate approach adds interpretive value beyond bivariate comparisons, as it controls for potential confounding factors. Although no formal interactions were tested due to sample size, the categorization of experience levels provided preliminary stratified insight.

In addition, our study shows that women tend to perceive a greater improvement in psychological and social well-being compared to men by practicing CrossFit^®^ (e.g., stress reduction, improved sleep quality, and social relationships). This could be linked to research on exercise motivation, which suggests that women often seek training environments that foster social support and group motivation [34].

For instance, previous research demonstrated gender differences in exercise motivation. Men were primarily driven by intrinsic motivations such as strength, competition, and personal challenge, while women were more influenced by extrinsic factors, including weight control and physical appearance [35,36].

Self-efficacy, the belief in one’s ability to complete activities, is another key factor in motivation, with studies showing higher levels in males [37,38]. Men also tend to engage in more intense physical activity than women [39]. Based on this, gender appears to significantly impact exercise motivation, self-efficacy, and activity levels.

Previous studies reported significant gender differences emerged in stress management, challenge, social recognition, competition, weight control, appearance, strength and endurance, and agility [40]. Overall, women were more likely to emphasize motivations related to stress relief, weight management, and physical appearance. In particular, they placed significantly greater importance on appearance and stress management than men while assigning lower importance to challenge [41]. In contrast, men placed more value on motivations involving personal challenge, social approval, competition, physical strength, endurance, and agility. In addition, Coyne et al. examined the relationship between CrossFit participation and women’s body image and eating behaviors and found that women preferred the CrossFit environment over other exercise settings due to its sense of community, inclusiveness, and structured programming. However, the study does not compare these findings with a male sample [42].

Moreover, future investigations could explore how gender differences relate not only to physical outcomes but also to psychological dimensions, such as body image perception and the risk of exercise dependence in the CrossFit contest. These factors are influenced by the type of sport participation and the level of physical activity engagement [43,44]. Examining these variables in the context of CrossFit^®^, which has not yet been thoroughly investigated, may provide additional insights into mental health and behavioral adaptations in male and female athletes. Although there are no significant differences between men and women in strength and endurance improvements (as indicated by our data on 1RM back squat and 2 km row performances), previous studies have found that women may have greater fatigue resistance in strength exercises than men, which could explain their lower injury incidence [45]. Additionally, chronic hormonal and immune responses to CrossFit^®^ training appear to differ between men and women, with men experiencing increased testosterone levels and reduced cortisol, while women maintain a lower testosterone-to-cortisol ratio throughout training [15].

Finally, future research should further analyze gender differences in CrossFit^®^, evaluating parameters such as post-workout recovery, metabolic adaptations, and injury prevention strategies tailored to each gender. For example, studies have shown that while men generally perform better in absolute power and strength-based CrossFit^®^ workouts, women tend to improve at a faster rate in repeated CrossFit^®^ Open workouts [34]. Understanding these dynamics could help optimize training programs for both genders.

The study has several limitations that may have affected the results and their interpretation. The exclusive use of electronic means for the administration of applications is one. While this methodology is convenient and quick, it can reduce the quality of responses compared to a face-to-face interview, where it would have been possible to clarify any ambiguities or elaborate on certain answers. In addition, electronic administration may have excluded some individuals who do not have regular access to the Internet or who do not feel comfortable using digital tools, thus introducing an additional selection bias. Finally, the study’s results inevitably depend on the subjective perceptions of the participants and their interpretations of the questions. It is essential to consider that although we have followed the guidelines for Questionnaire Development, the questionnaire has not been validated. Furthermore, within the questionnaire, there are no parts previously validated in other studies. Also, the lack of a reliability assessment method, such as the test–retest procedure, is one important limitation. Even if only a subjective view of the topic is provided, future studies could investigate this topic, starting from our findings, with another type of method or a reliable or validated questionnaire. The variability in interpretations may have introduced an additional source of error, as not all participants may have understood the questions in the same way or with the same level of depth. The entire questionnaire is based on the honesty of the participants and their accuracy in the answers, but we cannot be sure if their replies correspond to reality. Future studies should focalize the attention on the gender gap and, eventually, collect data on the injury rate and type, collecting more detailed information on their anthropometric characteristics directly in the field with objective data.

## 5. Conclusions

In conclusion, CrossFit^®^ athletes have a positive perception of this discipline, with no significant differences in responses between males and females except for the incidence in injuries. CrossFit^®^ positively affects physical conditions and psychological and social well-being, highlighting low exposure to injuries and high adherence to training sessions. However, these results should be viewed critically given the study’s limitations, particularly the unvalidated questionnaire and the lack of objective measures for performance and injury data.

## Figures and Tables

**Table 1:** Anthropometric and sociodemographic information of participants by gender. The table shows differences in the sample characteristics for continuous (**a**) and nominal (**b**) variables. F: female; M: male; n: number; SD: standard deviation; d/w: days per week.

(a)						
	F (Mean ± SD)	M (Mean ± SD)	U Mann–Whitney	*p*-Value		
age (year)	33.85 ± 9.9	34.70 ± 10.2	4841	0.55		
weight (kg)	61.45 ± 8	81.20 ± 9.3	534	<0.001		
height (cm)	162.73 ± 6.8	176.51 ± 5.6	638	<0.001		
BMI (kg/m^2^)	23.18 ± 2.4	26.03 ± 2.3	1962	<0.001		
weekly training frequency (d/w)	4.17 ± 1.1	4.42 ± 1.2	4508	0.15		
session duration (hours)	1.5 ± 0.7	1.68 ± 0.9	4612	0.20		
housework activities (hours)	1.5 ± 1.1	1.12 ± 0.9	3876	0.00		
**(b)**						
	**Sedentary Job (F-M)**	**Standing Job (F-M)**	**Manual Job** **(F-M)**	**Heavy Manual Job (F-M)**	**X^2^**	***p*-Value**
How would you define your work activity by the physical exertion involved? (n of participant)	50–49	38–42	8–11	0–4	4.20	0.241
	yes (F-M)	no (F-M)				
Have you ever participated in official CrossFit competitions? (n of participant)	35–51	61–55			2.80	0.094
	6 months (F-M)	12 months (F-M)	24 months (F-M)	more than 24 months (F-M)		
How many months have you been doing CrossFit? (n of participant)	13–13	6–10	14–5	63–78	6.38	0.095

**Table 2:** Number of answers and percentage for each option for the entire sample.

Questions	Answers Options			
	Yes, Considerably	Yes, Moderately	Yes, Slightly	No
1. Have you noticed an improvement in your physical strength since you started practicing CrossFit?	164 (81.2%)	(38) 18.8%	/	/
2. Have you lost weight or body fat since you started CrossFit?	73 (36.1%)	65 (32.2%)	42 (20.8%)	22 (10.9%)
3. Have you changed your eating habits since you practiced CrossFit?	102 (50.5%)	54 (26.7%)	21(10.4%)	25 (12.4%)
4. Did CrossFit reduce your stress level?	87 (43.1%)	76 (37.6%)	24 (11.9%)	15 (7.4%)
5. Did CrossFit practicing improve your sleep quality (REM phase and deep sleep)?	41 (20.3%)	78 (38.6%)	29 (14.4%)	54 (26.7%)
6. Did CrossFit practicing improve your self-esteem?	101 (50%)	67 (33.2%)	23 (11.4%)	11 (5.4%)
7. Did CrossFit practicing improve your daily energy?	91 (45%)	80 (39.6%)	21 (10.4%)	10 (5%)
	Scales	Bioimpedance analysis	Plicometry	Other
8. Have you rate the weight loss that justifies the previous answer? If so, which ones?	74 (40%)	60 (32.4%)	31 (16.8%)	20 (10.8%)
	No injuries	Injuries		
9. Have you suffered any injuries because of practicing CrossFit? If so, which ones?	125 (61.9%)	77 (38.1%)		
10. Has CrossFit influenced your social relationships?	Yes. in a positive way	Yes. in a negative way	No impact	
	160 (79.2%)	5 (2.5%)	37 (18.3%)	
11. Has CrossFit affected your productivity at work or in your studies?	125 (61.9%)	8 (4%)	69 (34.2%)	
	Yes	No	Maybe	
12. Have you seen any improvement in the 1RM back squat?	191 (96%)	8 (4%)		
13. Have you seen any improvements in the 2 km row?	168 (83.2%)	34 (16.8%)		
14. Have you made new friends or met new people through CrossFit?	199 (98.5%)	3 (1.5%)		
15. Would you recommend CrossFit to other people?	194 (96%)	1 (0.5%)	7 (3.5%)	

## Data Availability

The data presented in this study are available on request from the corresponding author due to privacy or ethical restrictions.
